# iPSC-Derived Retinal Pigment Epithelium Allografts Do Not Elicit Detrimental Effects in Rats: A Follow-Up Study

**DOI:** 10.1155/2016/8470263

**Published:** 2016-01-05

**Authors:** Peter D. Westenskow, Felicitas Bucher, Stephen Bravo, Toshihide Kurihara, Daniel Feitelberg, Liliana P. Paris, Edith Aguilar, Jonathan H. Lin, Martin Friedlander

**Affiliations:** ^1^Department of Cell and Molecular Biology, The Scripps Research Institute, La Jolla, CA 92037, USA; ^2^The Lowy Medical Research Institute, La Jolla, CA 92037, USA; ^3^Department of Pathology, VA San Diego Healthcare System, University of California San Diego, La Jolla, CA 92037, USA

## Abstract

Phototransduction is accomplished in the retina by photoreceptor neurons and retinal pigment epithelium (RPE) cells. Photoreceptors rely heavily on the RPE, and death or dysfunction of RPE is characteristic of age-related macular degeneration (AMD), a very common neurodegenerative disease for which no cure exists. RPE replacement is a promising therapeutic intervention for AMD, and large numbers of RPE cells can be generated from pluripotent stem cells. However, questions persist regarding iPSC-derived RPE (iPS-RPE) viability, immunogenicity, and tumorigenesis potential. We showed previously that iPS-RPE prevent photoreceptor atrophy in dystrophic rats up until 24 weeks after implantation. In this follow-up study, we longitudinally monitored the *same implanted iPS-RPE, in the same animals*. We observed no gross abnormalities in the eyes, livers, spleens, brains, and blood in aging rats with iPSC-RPE grafts. iPS-RPE cells that integrated into the subretinal space outlived the photoreceptors and survived for as long as 2 1/2 years while nonintegrating RPE cells were ingested by host macrophages. Both populations could be distinguished using immunohistochemistry and electron microscopy. iPSC-RPE could be isolated from the grafts and maintained in culture; these cells also phagocytosed isolated photoreceptor outer segments. We conclude that iPS-RPE grafts remain viable and do not induce any obvious associated pathological changes.

## 1. Introduction

The diverse functions of retinal pigment epithelium (RPE) cells are essential for photoreceptor activity [1]. RPE death or dysfunction results in photoreceptor degeneration characteristic of age-related macular degeneration (AMD), the leading cause of vision loss in the elderly [2]. While no cure exists, multiple studies performed in animal models of retinal degeneration and human subjects have provided encouraging evidence that RPE cell replacement prevents photoreceptor atrophy [3–14]. Multiple independent labs have shown that large numbers of RPE cells can readily be derived from stem cells (SC-RPE—for review see [15]). The use of autologous induced pluripotent stem cells (iPSCs) rather than embryonic stem cells (ESCs) may be advantageous since the risk of graft rejection may be reduced or obviated [16–18].

Other groups and ours have characterized SC-RPE using multiple methods. These studies have shown that SC-RPE strongly resemble primary RPE based on morphological, functional, transcriptomic, proteomic, and metabolomic analyses, and, most importantly, they function in vivo to significantly slow retinal degeneration [4, 6–8, 11, 14, 19–24]. With respect to their clinical application, however, there are unresolved concerns including safety and long-term viability of the hES- and iPS-RPE grafts [4, 6, 8, 10, 25]. Evidence from preclinical and early phase clinical trials has shown no adverse ocular effects in rodents or patients with hiPS-RPE or hESC-RPE allografts [9, 10, 13, 26–28], but histopathological exams of extraocular tissues have not been completely cataloged. If RPE grafts were short-lived or if they lost functionality over time, multiple subretinal injections, which involve transient macula-off retinal detachment and other risks, would be required for treating the slow and progressive degeneration characteristic of AMD. In carefully controlled experiments involving Cynomolgus Macaque and in human subjects, full visual recovery after macula-off detachments does not occur for roughly 3 months and in 6 months to 1 year after reattachment, respectively [29, 30].

In this study we continued monitoring the same iPS-RPE engrafted animals from our previous report [8] to catalog any gross detrimental ocular and/or systemic effects. In addition, we examined the viability and phagocytic function of the iPS-RPE in aging Royal College of Surgeons (RCS) rats.

## 2. Methods and Materials

### 2.1. Animal Studies

A mutation in the* MerTK* gene renders RPE cells incapable of phagocytosis and induces retinal degeneration in RCS albino rats [31]. Albino strains were used to facilitate identification of pigmented RPE cells. Subretinal injections of the three-week-old rats examined in this study and immunosuppression treatments were described previously [8]; injections were performed using protocols outlined previously in strict accordance with ethical guidelines of TSRI [32]. In brief, single RPE cells suspended in PBS were injected in a 0.5 *μ*L volume into the subretinal space. Pathologists and support staff at TSRI, UCSD (Department of Pathology), IDEXX RADIL, and Antech Diagnostics performed necropsies, analyzed selected tissues, and performed blood-work using standard protocols.

### 2.2. iPS Generation and RPE Differentiation

iPSCs were reprogrammed from somatic cells either using* OCT4* and small molecules (1F-iPS) [33] or with episomal vectors (EiPS) [34] and iPS-RPE were generated from both lines using directed differentiation as demonstrated in a multimedia protocol [35].

### 2.3. Cytology

Our established protocols for isolating and culturing RPE, immunocytochemistry, and immunohistochemistry have been described previously [8, 34]. Primary cultures were performed as described previously [8] from eyes from 2-year-1-month-, 2-year-2-month-, and 2-year-5-month-old albino RCS rats injected with iPS-RPE. The phagocytosis assay was performed as described previously [34] and briefly as follows: untreated cells were used to obtain baseline fluorescence. Cells were challenged with FITC labeled outer segments and incubated at 37°C for five hours. Cells were then rinsed with 1X Dulbecco's PBS and treated with trypsin (TrypLE; Invitrogen) for 5 to 8 minutes to detach cells and release bound outer segments. DMEM/F12 (Invitrogen) containing 2% FBS (Invitrogen) was added and samples were diluted 1 : 1 with FACS staining buffer (BD) containing DRAQ5 (1 : 2500; Cell Signaling) to distinguish cells from debris and outer segments. Samples were analyzed using a FACSCanto flow cytometer (BD) and gated based on DRAQ5 labeling. 5000 events were collected per sample. Data were analyzed using FlowJo software (version 8.8.6; Tree Star, Inc., Ashland, OR).

### 2.4. Transmission Electron Microscopy

For transmission electron microscopy, eye cups were fixed in 4% paraformaldehyde plus 1.5% glutaraldehyde in 0.1 M sodium cacodylate buffer overnight at 4°C followed by rinsing in 0.1 M Na cacodylate buffer for 1 h. Eyecups were postfixed in 1% osmium tetroxide in 0.1 M sodium cacodylate buffer for 2 h and then dehydrated in graded ethanol solutions. The tissues were incubated overnight in a 1 : 2 mixture of propylene oxide and Epon/Araldite (Sigma-Aldrich) and placed in 100% resin followed by embedding. The blocks were sectioned and used for high-magnification electron microscopy analysis. For immunohistochemistry, tissues were processed using standardized protocols [8]. Anti-Tra-I-85 (Abcam) [20], anti-OTX2 (R&D Systems), and anti-Iba1 (Wako) antibodies were used.

## 3. Results

A review of the daily monitoring reports from our colony revealed no obvious health or behavioral differences between iPS-RPE treated and untreated animals (SI Table 1 available online at http://dx.doi.org/10.1155/2016/8470263). There were also no significant differences between the weights of the uninjected, PBS injected, or iPS-RPE injected RCS rats (data not shown). Necropsies were performed on a subset of the animals with iPS-RPE implants and blood, eyes, brain, liver, and spleens were removed for more rigorous inspections (*n* = 10). Pathologists noted only aging-related phenomena including moderate splenic hemosiderosis, scattered (and mild) accumulations of hemosiderin- or lipofuscin-laden macrophages in some of the livers, and mammary tissues that appeared granular with pockets of white and occasional pale yellow fluids.

The blood test results also revealed no unusual abnormalities and were largely within normal reference levels (Table 1). White blood cell counts and mean corpuscle hemoglobin concentration levels were very mildly attenuated, while hemocrit levels were very slightly elevated. Slight polychromasia was seen in 4/7 male rats and, in one of those, moderate anisocytosis was evident. In general the erythrocytes were slightly normocytic and borderline hypochromic. All of these changes are more consistent with aging and unlikely to be due to chronic inflammation associated with the allografts.

**Figure 1:**
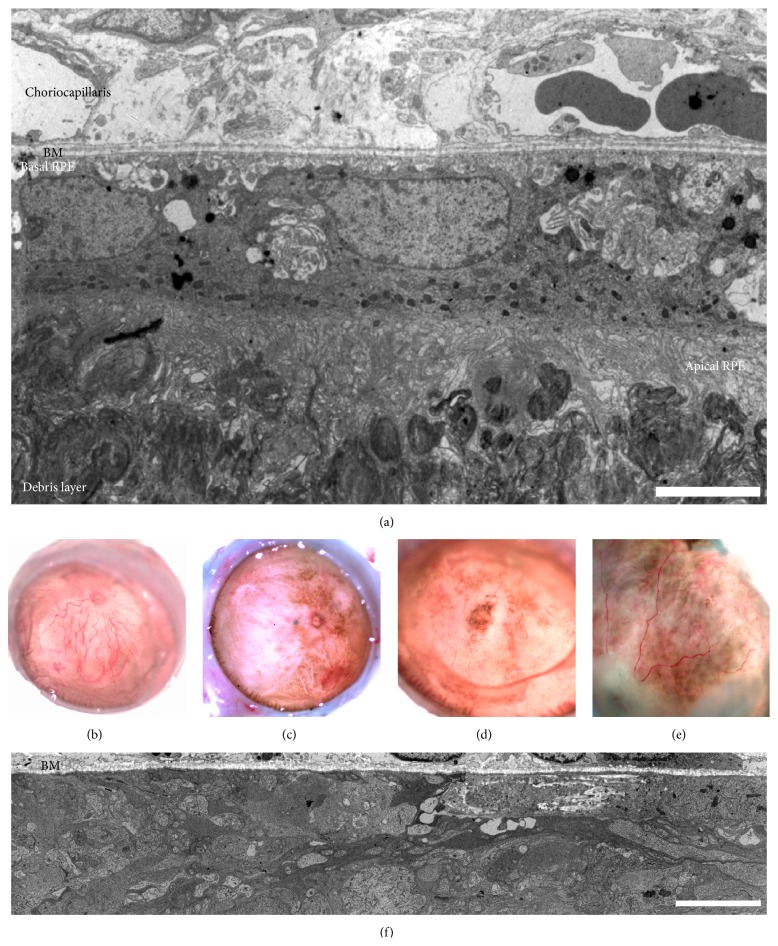
Melanin-laden cells are enriched in RPE compartments of aging RCS rat retinas. (a) Ultrastructural examinations from a RCS rat four weeks after implantation with iPS-RPE. Note RPE polarization between the choriocapillaris, Bruch's membrane (BM), and the debris layer. (b–e) A panel of 2+-year-old uninjected (b) and iPSC-RPE injected (c–e) eyes with corneas and lenses removed. Note the broad distributions of pigmented zones. (f) Electron micrograph of the subretinal space of a 17-month-old uninjected RCS rat. Scale bars = 5 *μ*m.

**Figure 2:**
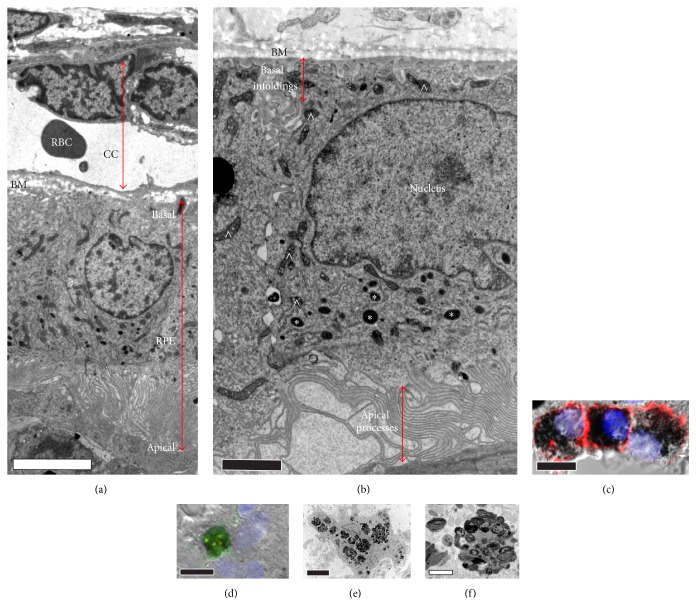
Cells with features characteristic of RPE are readily distinguishable in old RCS rats using ultrastructural examinations. (a and b) Ultrastructural RPE-like features are seen in electron micrographs. Just beneath the choriocapillaris (CC) lies Bruch's membrane (BM). Basal infoldings, melanosomes (marked with “*∗*”s; not to be confused with electron dense mitochondria (marked with “∧”s)), and long apical processes seen in normal RPE cells are apparent. (c) Immunohistochemistry performed on paraffin embedded sections was performed with anti-Tra-I-85 (which recognizes human cells) and anti-Iba1 (which recognizes activated microglia) antibodies. Membranes of pigmented cells in the RPE domain of the RCS retina are Tra-I-85 positive (red) and Iba1 negative (green). (d) Other pigmented cells outside of the subretinal space are probably macrophages based on Tra-I-85 negative (red) and Iba1 positive (green) signatures. (e) An electron micrograph of a melanin-laden macrophage. (f) Melanin in the macrophages accumulated in very dense aggregates in the phagolysosomes compared with the pigment distribution in RPE cells (b). Images are representative; evidence of proper integration and some unintegrated RPE (especially around the injection site) were seen in all iPS-RPE injected eyes examined. Scale bars: (a) = 5 *μ*m, (b) = 2 *μ*m, (c) = 10 *μ*m, (d) = 10 *μ*m, (e) = 5 *μ*m, and (f) = 1 *μ*m.

Ultrastructural examinations reveal that iPS-RPE injected as a suspension of cells can integrate into the subretinal space (Figure 1(a)). Examining single implanted cells at this magnification is useful for identifying subcellular structures including melanosomes (host albino cells have no melanosomes), basal infoldings, and long apical extensions, all characteristic of mature polarized RPE. After as late as 2.5 years after implantation, pigmented areas resembling grafted RPE can still be observed under the retina of enucleated eyes (Figures 1(b)–1(e)) compared with control uninjected regions (Figure 1(f)). Aging iPS-RPE grafts retain characteristics and features typical of RPE cells (Figures 2(a) and 2(b)). In addition, immunohistochemistry assays revealed that anti-human Tra-I-85 antibodies decorated the outer surface of the pigmented cells in the subretinal space (Figure 2(d)). These same cells were not immunoreactive for Iba1, a marker of activated microglia, thereby providing additional evidence that the cells are human iPS-RPE.

Conversely, Iba1 positive and Tra-I-85 negative melanin-laden cells were abundant in scattered patterns through the retina, but not in RPE domains. This lack of overlap of Iba1 and Tra-I-85 signals strongly suggests that these cells are host macrophages that ingested displaced human RPE cells (Figures 2(d)–2(e)). The survival potential for iPS-RPE in the subretinal space is notable, but since no ERG responses were detectable 18 weeks after injection (data not shown), these data suggest that iPS-RPE can provide only limited rescue in RCS rat retinas.

**Figure 3:**
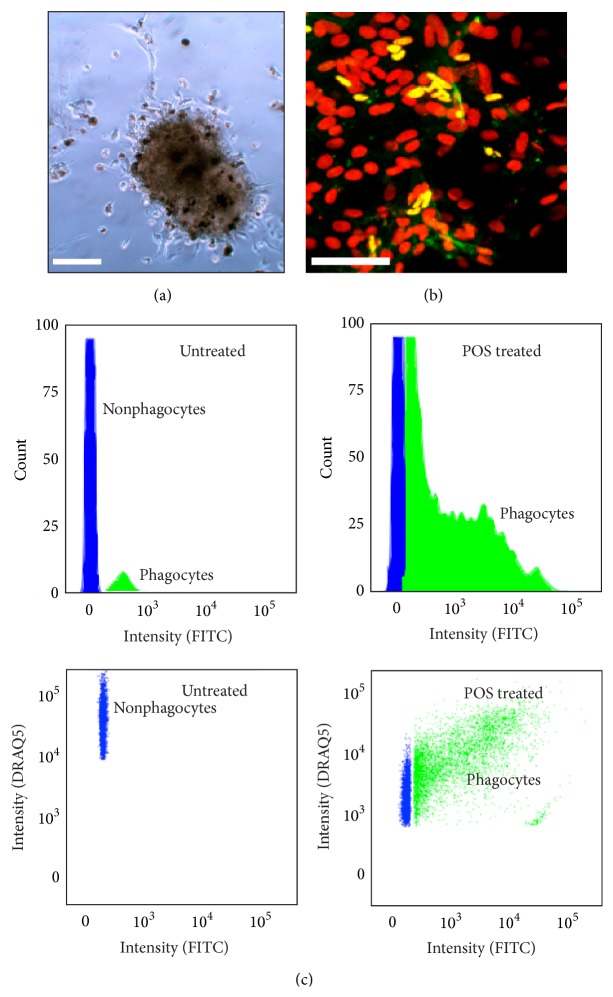
Data from immunocytochemistry and flow cytometry-based phagocytosis assays further suggest that the pigmented cells in the SRS are human RPE. (a and b) Pigmented clumps of cells from the subretinal space were isolated using RPE primary culture techniques (a). Some of the isolated cells are human RPE (labeled with antibodies that only recognize human OTX2—a molecular marker for RPE; DAPI = red; OTX2 = green (b)). (c) Some of the isolated cells internalized FITC-labeled porcine outer segments after five hours (labeled as phagocytes; green) in histograms (top panels) and scatter plots (bottom panels). DRAQ5 is a nuclear marker for living cells. Scale bars = 100 *μ*m.

Additional evidence that these are human RPE, and not host macrophages, is that they could be isolated from old animals, be maintained in culture, and phagocytose photoreceptor outer segments with previously established RPE binding/internalization parameters [36]. RPE primary cultures were generated from the eyes of 2.1-, 2.2-, and 2.5-year-old RCS rats (Figure 3(a)). The already existing challenges of generating pure primary RPE cultures are greatly exacerbated in old albino RCS rats since multiple non-RPE cells types and blood vessels populate the subretinal space [37]. Immunocytochemistry analyses with an antibody that only recognizes human OTX2, a molecular RPE marker, revealed that some of the isolated cells were human RPE (Figure 3(b)). When challenged with isolated porcine outer segments, some of the cells bound and internalized them in a manner consistent with RPE phagocytosis dynamics (Figure 3(c)) [34, 36]. This result strongly suggests that the 2+-year-old iPSC-RPE grafted cells are stable in the subretinal space and maintain phagocytic capacity even during retinal remodeling associated with retinal degeneration [38].

## 4. Discussion

Using iPSC technology, it is possible to generate large numbers of autologous RPE cells for transplantation that strongly resemble primary human RPE. In our previous study we showed that iPS-RPE provide transient anatomical and functional rescue in RCS rats [8]. In this study we were able to continue monitoring the* same* animals through the duration of their lives in order to determine the long-term efficacy and safety of RPE cell replacement therapy. By examining the viability and function of the same cells in the same animals both in our 2012 study and in this one, we draw the following conclusions: (a) hiPS-RPE that integrate into the subretinal space can provide transient neurotrophism (up to 18 weeks after injection) for photoreceptors. Nonintegrating cells can be cleared by host macrophages, even in immunocompromised states, without inducing features of chronic inflammation. This data is supported through evidence gathered using histology, optical coherence tomography, and focal ERG in iPS-RPE implanted eyes. (b) Implanted iPS-RPE outlive the photoreceptors, retain phagocytic function, and persist in the subretinal space during the entire course of the animals' lives. (c) Based on the parameters examined in this study, we report no obvious complications due to exogenous RPE delivery, as long as the cells integrate properly. (d) Abnormalities observed were due to cell reflux and other complications from the subretinal injections (SI Figure 1). These complications are likely to be minimized since the subretinal injections will be performed by skilled vitreoretinal surgeons in much bigger eyes using optimized protocols [13, 39].

Reports indicate that stem cell-derived RPE survive for only 13 weeks and as long as >220 days in the subretinal space [4, 6, 10]. Furthermore, implanted RPE cells can form aggregates and double-layered structures with iPSC-RPE and host RPE [4, 6, 9, 10]. Other groups are now focused on culturing RPE cells either as intact sheets or on synthetic substrates to facilitate delivery of intact monolayers. Evidence suggests that RPE delivered using this method may enhance stability of the grafts, but this approach does not ultimately seem to prolong the photoreceptor rescue effects [25] and involves a more complicated surgery. Of the 31 eyes examined in this study, iPS-RPE were fundoscopically detectable in all of them after delivering the cell in suspension, and histological examinations revealed the presence of integrated and unintegrated (primarily directly around the injection site) cells. Ultimately, the integrated cells exhibited polarity and pigmentation characteristic of intact RPE monolayers and survived for as long as two and a half years. Therefore, both approaches have their own advantages and limitations, and the decision to implant RPE cells in suspension or on scaffolds may require case-by-case decisions based on the integrity of Bruch's membrane.

## 5. Conclusion

In summary we see no evidence of abnormal RPE cell migration and no evidence of tumorigenesis or chronic inflammation up to 2 1/2 years after implantation. iPSC-RPE injected as a cell suspension can stably integrate into the retina and, despite the fact that they cannot provide permanent photoreceptor rescue, retain phagocytic function and are observed throughout the lives of the rats without eliciting gross detrimental effects. This study confirms findings from other groups that RPE transplantation does not likely represent a cure [9, 10]. However, based on our previous and current findings, RPE cell transplantation can provide significant therapeutic benefits without inducing off-target deleterious effects.

## Supplementary Material

A table (SI Table 1) summarizing the daily health and behavior reports for the rats involved in this study and images showing occasional complications involved with the subretinal injections (SI Figure 1) are included in the supplemental material.

## Figures and Tables

**(a):** 

Sex	Age	WBC	RBC	HGB	HCT	MCV	MCH	MCHC	RBC Morph.
M	1 y 10 m	5.3	8.3	18.3	52	63	19.7	31	Normal
M	1 y 10 m	5.9	8.6	16.8	52	60	19.6	32	PC^*∗*^
M	1 y 5 m	8.7	7.6	14.8	49	64	19.4	30	PC^*∗*^
M	1 y 5 m	4.5	8.8	16.4	53	60	18.7	31	Normal
M	1 y 5 m	4.5	8.7	16.3	52	60	18.7	31	PC^*∗*^
M	1 y 5 m	7	8.9	16.7	55	62	18.8	30	PC^*∗*^/AN^*∗*^
F	1 y 6 m	3.7	7.5	14.2	47	63	18.9	30	Normal
M	1 y 6 m	5.4	8.3	15.6	52	62	18.7	30	Normal
F	1 y 5 m	4.8	7.8	15	49	63	19.3	31	Normal
F	1 y 5 m	3.7	7.1	13.7	46	65	19.3	30	Normal

Ave.		5.4	8.2	15.8	51	62	19.1	31	n/a
Ref. range		5.5–11	5.5–10.5	10.6–17.2	33–50	47–66	15–23	31–38	n/a
Units		10^3^/*μ*L	10^6^/*μ*L	g/dL	%	fL	pg	g/dL	n/a

**(b):** 

Sex	Age	Platelet Ct.	Platelet Est.	Neutrophils	Lymphocytes	Monocytes	Eosinophils
M	1 y 10 m	1101	Increased	1060 (20%)	4028 (76%)	106 (2%)	106 (2%)
M	1 y 10 m	377	Adequate	2537 (43%)	3127 (53%)	118 (2%)	118 (2%)
M	1 y 5 m	1251	Increased	5133 (59%)	3306 (38%)	174 (2%)	87 (1%)
M	1 y 5 m	1133	Increased	945 (21%)	3465 (77%)	45 (1%)	45 (1%)
M	1 y 5 m	1176	Increased	990 (22%)	3330 (74%)	45 (1%)	90 (2%)
M	1 y 5 m	1110	Adequate	2450 (35%)	4340 (62%)	70 (1%)	140 (2%)
F	1 y 6 m	1038	Adequate	1147 (31%)	2405 (65%)	74 (2%)	74 (2%)
M	1 y 6 m	1242	Adequate	1674 (31%)	3510 (65%)	108 (2%)	108 (2%)
F	1 y 5 m	817	Adequate	1632 (34%)	3072 (64%)	48 (1%)	48 (1%)
F	1 y 5 m	667	Adequate	925 (25%)	2664 (72%)	37 (1%)	74 (2%)

Units		10^3^/*μ*L	/*μ*L	/*μ*L	/*μ*L	/*μ*L	/*μ*L
