# Supplementary material for: iPSC-Derived Retinal Pigment Epithelium Allografts Do Not Elicit Detrimental Effects in Rats: A Follow-Up Study
Source: Stem Cells Int. 2016 Jan 5;2016:8470263. doi: 10.1155/2016/8470263 (PMC4736415; doi:10.1155/2016/8470263)
Supplement: Supplementary file 1 — A table (SI Table 1) summarizing the daily health and behavior reports for the rats involved in this study and images showing occasional complications involved with the subretinal injections (SI Figure 1) are included in the supplemental material. [file 8470263.f1.pdf]

Uninjected			
**Age**	**Sex**	**Observation**	**Location**
0 y 4 m	Female	Alopecia/Pruritus/Dermatitis	back
0 y 4 m	Female	Alopecia/Pruritus/Dermatitis	rear dorsum
0 y 5 m	Female	Eye closed/discharge	right eye
0 y 5 m	Female	Eye closed/discharge	right eye
0 y 6 m	Female	Eye closed/discharge	left eye
0 y 6 m	Male	Found Dead	
0 y 7 m	Female	Neoplasm	by front limbs
0 y 7 m	Male	Not Drinking/Dehydrated	
0 y 11 m	Female	Eye closed/discharge	right eye
0 y 11 m	Female	Eye closed/discharge	left eye
1 y 0 m	Female	Found Dead	
1 y 1 m	Female	Lame/Lethargic/Unthrifty	right forelimb
1 y 3 m	Female	Neoplasm	inguinal-left side
1 y 4 m	Female	Neoplasm	abdominal area
iPSC-RPE injected			
**Age**	**Sex**	**Observation**	**Location**
0 y 3 m	Female	Lesions--cut, gash, wound, etc.	left side
1 y 0 m	Female	Eye closed/discharge	left (uninj) eye
1 y 4 m	Female	Neoplasm	axillary-right side
1 y 4 m	Female	Hunched/Ruffled Coat/labored breathing/weight loss	
1 y 5 m	Male	Swollen foot	left rear foot
1 y 5 m	Female	Eye closed/discharge	left (uninj) eye
1 y 5 m	Female	Eye closed/discharge	right (inj) eye
1 y 10 m	Female	Eye closed/discharge	left (uninj) eye
2 y 1 m	Male	Porphyrin	left (uninj) eye

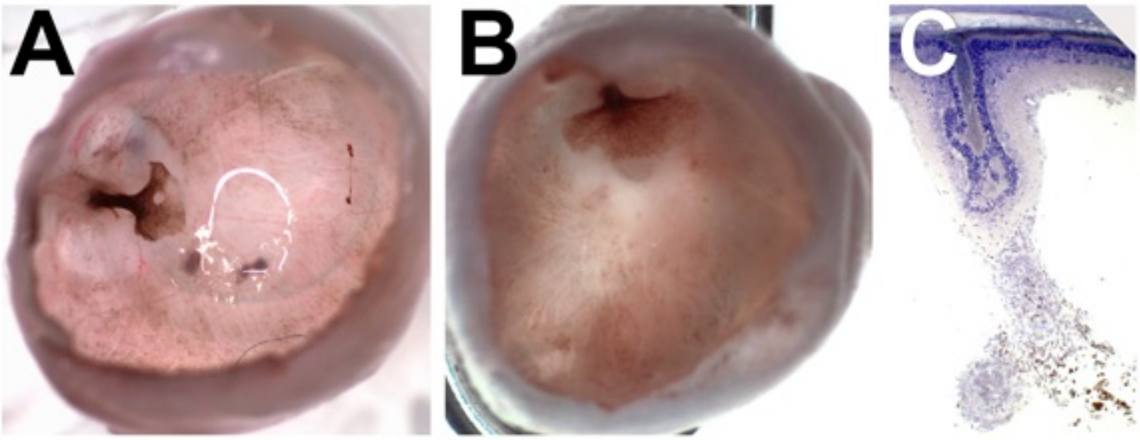

SI Figure 1

SI Table 1. **Summary of the filed health and daily behavior reports**. All the reports were collected and listed by age.

SI Fig. 1. Occasional complications of injecting suspensions of iPS-RPE. (A-C) Reflux of RPE cells and acute damage to the retina at the injection site. (A&B) Pigmented cells can be seen in a “spout” in the vitreous of enucleated eyes. (C) Histology shows damage at the injected site and accumulations of pigment-laden cells in the vitreous.
